# Exhaustion in Healthcare Workers after the First Three Waves of the COVID-19 Pandemic

**DOI:** 10.3390/ijerph19148871

**Published:** 2022-07-21

**Authors:** Marina Ruxandra Oțelea, Agripina Rașcu, Cătălin Staicu, Lavinia Călugăreanu, Mădălina Ipate, Silvia Teodorescu, Ovidiu Persecă, Angelica Voinoiu, Andra Neamțu, Violeta Calotă, Dana Mateș

**Affiliations:** 1 Clinical Department 5, University of Medicine and Pharmacy Carol Davila, 050474 Bucharest, Romania; marina.otelea@umfcd.ro (M.R.O.); agripina.rascu@umfcd.ro (A.R.); 2 Clinic of Occupational Medicine, Colentina Clinical Hospital, 020125 Bucharest, Romania; 3 National Center for Monitoring Community Risks, National Institute of Public Health, 050463 Bucharest, Romania; catalin.staicu@insp.gov.ro (C.S.); lavinia.calugareanu@insp.gov.ro (L.C.); silvia.teodorescu@insp.gov.ro (S.T.); angelica.voinoiu@insp.gov.ro (A.V.); andra.neamtu@insp.gov.ro (A.N.); dana.mates@insp.gov.ro (D.M.); 4 Regional Center of Public Health, National Institute of Public Health, 700465 Iași, Romania; madalina.ipate@insp.gov.ro; 5 Regional Center for Public Health, National Institute of Public Health, 400349 Cluj Napoca, Romania; ovidiu.perseca@insp.gov.ro

**Keywords:** exhaustion, healthcare workers, COVID-19, effort/reward score, overcommitment

## Abstract

This study aims to identify the determinants of exhaustion of frontline and second-line healthcare workers (HCW) during the third wave of the COVID-19 pandemic. A case–control study was conducted based on an anonymously distributed questionnaire, which was completed by 1872 HCW. Exhaustion was assessed with a validated Romanian questionnaire. The Siegrist questionnaire was used to determine workload, reward and overcommitment. Frontline HCW reported significantly more frequent longer working hours (*p* = 0.0009) and a better perception of the management of the risk for infection (*p* = 0.0002) than second-line HCW. The effort and overcommitment scores were higher in frontline HCW (9.51 + 1.98 vs. 8.45 + 21, *p* < 0.001 and 16.34 ± 2.80 vs. 15.24 ± 2.94, *p* < 0.001, respectively) and the reward scores were lower (5.21 ± 1.522 vs. 5.99 ± 1.44, *p* < 0.001). In the fully adjusted regression model, age, imbalance between effort and reward, overcommitment and management of the risk of infection in the workplace were associated with the exhaustion score in each category of HCW. The number of working hours was correlated with exhaustion in frontline HCW and occupation in second-line HCW. There were more similarities than differences between frontline and second-line HCW. Even if frontline HCW had a higher risk of exhaustion, the risk was not negligible for all HCW.

## 1. Introduction

The last two years have dramatically changed the way work is performed in almost all domains. For healthcare workers (HCW), major concerns started from the very beginning of the pandemic and some of them persist nowadays, with exacerbations during each COVID-19 wave. The sudden increase in the number of patients rapidly generated a high workload; the shortages in staff were materialized in longer working hours, more nightshifts and deployment to other services or even towns. Work did not change only in its quantitative aspect. The insufficient/lack of knowledge about COVID-19, the frequent changes in procedures, controversies about the suitable procedures and treatments that did not had enough time for proper validation, the ethical dilemmas due to the scarcity of resources, the frequent contact with severe/severely ill patients and the more frequent failure to treat them despite the enormous efforts created an unprecedented negative emotional impact, even among the highly motivated, which questioned their self-perception about the efficacy of their profession. The perceived personal risk of infection and the inadequate protective equipment, and the sufferance or death of colleagues and loved ones added supplementary emotional burden.

All these work-related factors assemble towards chronic workplace stress, the main determinant of burnout. The concept of burnout implies a crisis in the relation between the individual and the work that extends beyond tiredness towards hopelessness, discouragement and even alienation [1] and includes a mixture of psychological and physical symptoms. Burnout is considered the endpoint of a sequential and generally long process. Briefly, work stressors generate a psycho-social strain (manifested as exhaustion), which leads to defense mechanisms for coping (such as cynicism) ending up with a reduction in personal accomplishment and work satisfaction [2,3,4]. Thus, the first step to developing burnout [5] is the experience of content and context stressors in the job, such as concurrent high workload, particularly if the work generates negative emotions, and unmet expectations from the job. In acute stress, the consequence is fatigue, but if the recuperation is not possible (as it was the case during the COVID-19 pandemic), exhaustion will follow. Exhaustion, physical and emotional, has even been proposed as the unique core definition of burnout [6]. In fact, exhaustion seems to be the most important dimension associated with clinical diagnosis [7].

The stages of the pandemic had different consequences on the mental health of HCW. The third wave, to which we refer in this research, had some specificities. During this wave, the “pandemic fatigue”, defined by WHO “as demotivation to follow recommended protective behaviours, emerging gradually over time and affected by a number of emotions, experiences and perceptions” [8], was already manifest. For the Romanian healthcare system, it was the period in which the clinical procedures were better clarified, the protective equipment was available in a greater extent, but with considerable differences between the COVID-19 wards and the rest of the medical services. Paradoxically, with strict safety procedures, the rhythm/pace of shifts and the multidisciplinary teams in place in many hospital departments dedicated to COVID-19 patients, the workload and organizational context were better managed than in other departments or in outpatient clinics, where contact with potentially infected patients was unknown, as testing was far from sufficient and personal protective equipment was scarce. Dealing with patients exhausted by pandemic fatigue and their personal social and economic problems increased the burden of emotional stress for all HCW in direct contact with patients. The fact that testing was not generally available led to negative emotions derived from uncertainty. Several national studies support this statement, for example, hospital-based research found that burnout was more frequent in non-COVID-19 wards [9]. Another study, based on a large sample of general practitioners (GP) from almost all Romanian counties, emphasized the inefficient and insufficient support provided by the authorities to GPs, which generated a high level of stress, particularly in younger female GPs, those working in grouped medical offices and in regions characterized by a high score of two social dimensions, avoiding uncertainty and psychological collectivism [10].

The mental health impact during the pandemic has been assessed mainly in frontline HCW (flHCW), although the entire health system has been challenged. flHCW are generally defined as those dealing directly with COVID-19 patients. However, doctors, nurses and other healthcare professionals from laboratory services, public authorities, research and education organizations, who were not in direct contact with patients, also had a high pressure to deliver results that have an impact on the health of the population. They are included in the second-line HCW (slHCW) group. The responsibility, the uncertainties related to their decisions and time pressures were greatly manifested among these HCW as well. Although slHCW, as defined above, are included in some studies assessing work-related stress, the number is generally not sufficient for a proper, specific analysis of this group [11]. The level of psychological distress was significantly higher in the administrative staff of an acute care hospital compared to doctors [12], but post-traumatic stress disorder was present only in those in contact with COVID-19 patients [13].

Through this study, we intend to contribute to the understanding of the effect of this medical crisis in Romanian HCW, mainly to ascribe particular determinants of exhaustion for frontline and second-line HCW. The high-exhaustion latent burnout profile was associated with excessive workload [14]; therefore, we selected several objective (number of hours of work/day, night shifts and number of patients/day) and subjective (the effort component of the Siegrist model) workload measures as explanatory variables for exhaustion. The Siegrist reward dimension was also strongly related to exhaustion in previous studies on flHCW during the pandemic [15,16], but it has not been explored in slHCW.

The Siegrist model of work-related stress highlights an intrinsic factor influencing the balance between effort and reward, namely overcommitment [17]. Overcommitment represents the excessive engagement of the employee, their need for control and approval. It is a particular form of coping with work-related stress, which has been associated with various health issues [17], including exhaustion [18]. The attitude of HCW during a pandemic fluctuates between the duty and the fear to work, [19], which makes overcommitment relevant as a pre-requisite for work-related stress and exhaustion. Fear of infection was a major stress source during the first wave [20], but it has not completely disappeared.

In view of all of the above, we hypothesized that workload, the imbalance between effort and reward, overcommitment and the perception of risk (including personal infection by SARS-CoV2) are related to exhaustion in all HCW, being either in the frontline or second line.

## 2. Materials and Methods

### 2.1. Data Collection

This analysis is part of more comprehensive longitudinal study, which collects data about the occupational risk for COVID-19, the mental health and long-term consequences of COVID-19 in HCW together with the variation in the immune response in vaccinated HCW during the COVID-19 pandemic [21]. In brief, a questionnaire was anonymously distributed via the Survey Monkey platform to HCW from public and private medical settings, public medical institutions and local public health authorities, using their institutional email address. Email addresses were collected from the official website of the Public National Health Insurer (*Casa Națională de Asigurări de Sănătate*), which has contracts with the majority of healthcare providers, public or private, and from the web sites of the public institutions. There were 2698 persons who answered to the invitation; among them, 1872 (69.4%) responded to all items. An informed consent agreement for online qualitative research was provided to each participant, who had the option to accept or reject the participation before responding to the first question of the survey. No selection criteria were applied and all subjects who accepted to participate were included in the analysis. The specificity of this cohort is the mixture of HCW with activity directly related to patients and HCW involved in administrative, research and health education roles that do not provide, during their regular activities, any direct medical service to patients.

Within the cohort, a case–control study was conducted, selecting the participants having known or possible contact with COVID-19 patients, considered as flHCW (1311 HCW), whereas all HCW that did not provide direct services to patients were included in the slHCW group (561 HCW).

The current analysis included data regarding the mental health status in the enrolment phase, namely from May 2021–June 2021. This period of time corresponds to the steep decline in the number of COVID-19 cases, immediately after the third wave of the pandemic in Romania.

The study was approved by the Ethical Committee of the National Institute of Public Health in Romania.

### 2.2. Outcomes

Exhaustion was assessed with a five items scale, designed to explore the dimension of burnout from the Maslach Burnout Inventory, validated in Romania, and made available for researchers as part of a research project of the Faculty of Psychology and Educational Sciences of the University of Bucharest [22]. The response scale was 5 points for each of the 5 items and the score of exhaustion was obtained by adding up all items. The Cronbach’s alpha of the exhaustion score was 0.89. The exhaustion score was the dependent variable in the univariate and multivariate regression models. The scores for exhaustion that were >75% of the maximum possible score were classified as high risk for exhaustion in the binary regression models. Scores <75% were considered as the reference.

### 2.3. Explanatory Variables

A high workload, particularly under emotional stress, with unmet expectations from the job are occupational stressors related to exhaustion [5]. Therefore, we assessed the imbalance between high effort and reward with a validated patient-reported outcome, the Siegrist questionnaire [23] and with several objective occupational determinants, such as occupation, tenure, number of working hours, working schedule (e.g., night shifts) and number of patients/day. The Siegrist questionnaire also investigates overcommitment, defined as “a set of attitudes, behaviours and emotions that reflect excessive striving in combination with a strong desire of being approved and esteemed” [24]. The scores were calculated according to the methodology provided by the authors [23]. The Cronbach’s alpha for the effort score in our sample was 0.83, for the reward score was 0.86 and for the overcommitment 0.86.

The occupations were classified according to the international standard classification of the World Health Organization in four major categories: doctors, nurses, other health professionals and management and support personnel [25]. Among the HCW who responded to this questionnaire, 70.94% were doctors, 13.27% were nurses, 8.99% other healthcare professionals and 6.85% management and support personnel. The distribution was different in the two groups; as expected, in the flHCW group, doctors and nurses predominated (together forming 94.28% of the group). In the slHCW group, doctors and nurses represented only 66.61% of the total. The HCW were also asked to mention their medical institution affiliation.

Overall, 368 worked in hospitals, 850 in ambulatory services (among which 453 in family practice), 9 in emergency services, 48 in pharmacies, 503 in the central or local Public Health authorities, 31 in medical laboratories and 55 in research institutes or medical universities, and 9 in other unspecified medical organizations. The majority of HCW worked in hospitals (89.13%), ambulatory services (98.49%), emergency services (88.88%), dental services (92.72%) and pharmacies (89.58%), provided direct services to COVID-19 patients and were included in the flHCW group. The slHCW included the majority of staff from central and local public health authorities (83.98%), laboratory services (70.97%) and research and teaching roles (82.72%).

Four categories of tenure were defined: <1 year, 1–5 years, 6–10 years and >10 years.

One item of the questionnaire was dedicated to the “average duration of working hours during the pandemic”. The possible responses were based on the typical working schedules in the Romanian healthcare system: part-time (4 h), full-time for doctors (7 h), full-time for other HCW (8 h), shifts (12 h) and night shifts + continuous day schedule (>12 h).

Regarding working in shifts, we considered 3 possibilities of response: no shifts, day shifts or mixed (day and night shifts). It is important to note that, during the pandemic, part of the HCW from the public health authorities, classified in this study as slHCW not in direct contact with COVID-19 patients, were also working in night shifts, answering to incoming telephone calls from citizens.

In the flHCW group, we also investigated the number of patients examined during an average working day. In this item, the HCW could choose between <5 patients/day, between 5–15 patients/day and >15 patients/day.

As specific determinants of stress during the pandemic, we also considered in the analysis the personal occurrence of COVID-19 (present/absent and persistence of symptoms after acute illness) and the perceived management of the risk of infection in the workplace. This was assessed with the following question: “From your point of view, how was the risk for infection with SARS-CoV-2 managed at your workplace?” Responses were based on a five-point Likert scale (very well, well, acceptable, badly, and very badly).

Age, gender, marital status (single or living as a couple) and level of education (undergraduate and university degree) were considered in the analysis. The university degree category included respondents with university and post-university studies. All others (secondary school, vocational schools, etc.) were included in the undergraduate category.

### 2.4. Statistical Methods

Characteristics were summarized for the study population and for the two groups and presented as mean and standard deviation or medians. The numerical variables were initially checked for the normality of the distribution. Nominal variables were compared with Pearson’s chi-squared test and numerical variables with the Mann–Whitney test. The Kruskal–Wallis test was used in the comparison of more than three groups of variables. Regression was performed for the univariate analysis of the association between covariates and the outcomes. Coefficients and 95% confidence intervals (CIs) were calculated. The multivariate regression models were adjusted for variables showing a significant association in the univariate analysis. A *p*-value less than 0.05 indicates statistical significance. Data were processed with a STATA software (STATA MP Version13.0, College Station, TX, USA).

## 3. Results

### 3.1. Description of the Study Population

There were 1311 valid questionnaires in the flHCW group and 561 in the slHCW group.

The general characteristics of the study population are presented in Table 1.

The score of exhaustion of the entire sample was on average 14.35, with 4.73 of standard deviation. This score was significantly higher in flHCW (14.88 ± 4.65) compared to slHCW (13.12 ± 4.69), *p* < 0.001.

The number of flHCW with a high exhaustion score was 283 (21.59%); in the slHCW group, the high exhaustion score was found in 77 respondents (13.73% of the slHCW). The difference was statistically significant (*p* < 0.001).

### 3.2. Univariate Analysis

The results of the univariate analysis are presented in Table 2.

In the flHCW group, the univariate analysis showed that the frame of determinants related to exhaustion was composed of a mixture of individual (age, level of education and overcommitment) and objective job characteristics: occupation and workload (average number of working hours/day and number of patients/day). The organizational context was represented by the effort/reward score. Among the objective job characteristics, the number of patients/day was directly related to the exhaustion score and to the odds of a high risk score. There was no significant difference in the exhaustion score by occupational categories in flHCW (H = 6.17, *p* = 0.10). However, in the one-to-one category comparison, doctors had significant higher values than the respondents from the other healthcare professionals category (U = 30,670, *p* = 0.03).

In the slHCW group, the personal characteristics related to exhaustion were gender (women had a higher chance of exhaustion) and younger age. The exhaustion score was significantly different by occupation (H = 12.23, *p* = 0.007), namely the exhaustion score of the health management and support group was significantly lower than the exhaustion score of doctors (U = 8982, *p* = 0.0005) and the other health professionals groups (U = 4730.5, *p* = 0.008).

Among the occupational factors, working longer hours and night shifts and the effort/reward score were significantly related to the exhaustion score.

Characteristic to the pandemic were two additional components of this frame, the appraisal of how the infection risk was managed in the workplace (Table 1) and the personal history of COVID-19 (Table 1). Overall, subjects diagnosed with COVID-19 had significantly higher scores for exhaustion (coef. = 0.95, CI = 0.47–1.44, *p* = 0.0001). HCW infected with SARS-CoV2 had an OR = 1.42 (CI = 1.11–1.83, *p* = 0.006) for high risk of exhaustion. The persistence of symptoms was also associated with the high risk of exhaustion (OR = 1.65, CI = 1.07–2.56, *p* = 0.02).

### 3.3. Multivariate Analysis

For the flHCW group, the full saturated regression model included: age, level of education, occupation, average number of working hours/day, number of patients/day, the effort score, the reward score, the overcommitment score, the appreciation of how the infection risk was managed in the workplace and the personal history of COVID-19. Different regression models were tested according to the categories of the determinants (Table 3).

In the fully adjusted regression model, the high exhaustion risk of flHCW was related only to overcommitment, the imbalance between effort and reward and age (R^2^ = 0.26) (Table 4).

For the slHCW group, the full saturated regression model included: age, gender, level of education, occupation, average number of working hours/day, working in night shifts, the effort score, the reward score, the overcommitment score, the appreciation of how the infection risk was managed in the workplace and the personal history of COVID-19. The results of the different regression models are presented in Table 5.

After adjustment for gender, night shift and occupation, the high exhaustion score in slHCW was related to the overcommitment and the effort–reward imbalance and to the average working hours/day (R^2^ = 0.25) (Table 6).

### 3.4. Comparison of the Perceived Job Stressors and Exhaustion Score between flHCW and slHCW

The scores of effort and overcommitment were significantly higher, while the score of reward was significantly lower in the flHCW group compared to that of the slHCW (Table 1). The number of participants with effort/reward score imbalance (score higher than 1) was 817 (62.13%) in the flHCW and 195 (34.75%) in the slHCW groups (χ^2^ = 120.15, *p* < 0.0001).

The score of exhaustion in the two groups according to occupations is presented in Table 7. Significant higher values were noted in the comparison of doctors between the two groups.

More slHCW had a lower appreciation of the occupational risk management against infection (Table 1). Although the median of the score was identical (3), the average was higher in the flHCW group than the slHCW group and reflected this different perception (3.04 vs. 2.83, U = 22.01, *p* = 0.00001).

The number of HCW infected with COVID-19 was higher and the persistence of the symptoms after the acute episode was more frequent in the flHCW group (Table 1).

However, being diagnosed with COVID-19 increased the odds of a higher exhaustion score only in flHCW (Table 8).

## 4. Discussion

These are the results of a study on exhaustion in first- and second-line HCW after the third wave of the COVID-19 pandemic in Romania. The study was conducted in a healthcare system that is still poor according to European standards and covered a large spectrum of occupations in the medical sector, two distinctive characteristics that are approached rather infrequently in publications regarding the negative impact of the current pandemic in HCW [26]. There are also few studies that compare HCW directly providing medical services to patients with HCW performing other medical activities, and our data expand on the picture about the similarities and differences in HCW with various contributions to the healthcare system.

The prevention of exhaustion is important, particularly in periods of intense activity in the medical system, as are those during a pandemic. It seems that several influencers of exhaustion were common in flHCW and slHCW in the multivariate analysis: overcommitment, high imbalance between effort and reward, and age. With the exception of age all others are explored by the Siegrist questionnaire, which proved to be a valuable instrument to explore the risk of exhaustion.

For flHCW, the level of education and the perception of infectious risk management were also significant, while for slHCW, the occupation was a differential.

In the univariate analysis, a high exhaustion score was associated with occupation (doctors being the most affected), the number of working hours, management of the infection risk in the workplace and personal history of COVID-19 in both study groups. There were also distinctive associations: in flHCW, a high risk of exhaustion was also inversely related to age and directly related to a higher level of education, while in the slHCW group, it was associated with gender (more frequent in women) and night shift.

Overcommitment is a natural tendency of many HCW during a sanitary crisis, because the extra workload outpaces the supply of available staff. Our results provide arguments that this reaction is highly related to exhaustion, which might lead to negative professional results. This has been shown even before the pandemic. For example, in a study conducted in China in July 2019, high scores of overcommitment and imbalance between effort and reward were associated with exhaustion, but not with professional efficiency, in HCW [27]. During the pandemic, overcommitment and a high imbalance in effort–reward were associated with insomnia [28], anxiety and depression [15,29], all of them reducing the effectiveness and efficiency of the medical services. Even more, one fifth of people affected by exhaustion will change their job in the following years [30], creating additional problems for the healthcare system in the near future.

Based on our results, the Siegrist questionnaire could identify candidates for exhaustion. Both external elements (effort and reward) should be addressed by the organizational management. We did not investigate the efficacy of the different solutions that might be applied in the pandemic context. Referring strictly to our results, the work context seems to be significant, because the objective job characteristics lost their statistical significance after adjustments in the flHCW group. In slHCW, only the long working time remained significant. This job characteristic was, in fact, a more general issue in Romania, reported also in the context of other occupations [31]. Night shifts are not usual among slHCW and were introduced in response to the need of permanent surveillance activities related to the pandemic. This raises the point about a better transition to the new work schedule. This was suggested by another study [32] referring to medical personnel who was not used to onsite night shifts and had also difficulties in adapting. The coping strategy was forming teams with HCW that were already familiarized with night shifts and the results were favorable.

The intrinsic component of the Siegrist model, overcommitment, is an ineffective coping strategy to work pressure and not a solution. In contrast to workaholism, which reflects a high internal motivation and conscientiousness for work, leading to professional achievements and job satisfaction, overcommitment is driven by external factors (work content and context) and is related to neuroticism, low job satisfaction and burnout [33]. Therefore, interventions focused on the workload should be balanced with a personal approach. Strategies to develop a better work–life balance and self-care should be developed among these HCW to maintain their dedication and to avoid exhaustion.

Another common finding was the inverse relation between exhaustion and age. Younger age was found to be a determinant of exhaustion or even burnout in several studies referring to flHCW [34,35,36]. Occupational and non-occupational factors contribute to this relation. In the general working population, young people with less than one year in the current job were more affected by the pandemic [37]. Job changes are found to be a major stressor and this became even more visible during the pandemic [38]. We did not find any significant relation with tenure, but the items referring to tenure in our questionnaire explored only the total occupational experience and did not investigate the working period in the current workplace. On the other hand, the number of participants with less than 1 year of total experience was very low; therefore, we cannot conclude about this association.

There are many explanations as to why younger age had higher scores of exhaustion in our study. The acquisition of practical skills, knowledge and self-confidence in medical professions develops gradually with time. Young professionals need adequate training programs for their specialty and the stability and even the participation to the training programs was affected in many aspects during the pandemic [39]. As a result, resident doctors had exceptionally high scores of exhaustion [40]. In a balanced study sample, Canadian flHCW and second-line HCW of younger age had an increased risk of exhaustion [41]. In this study, having a child at home had a significant contribution to this risk.

The percentage of women in our sample was high (81.84%), and this might be considered a limitation of the study. However, this gender distribution reflects the statistical data of the Romanian Statistical Yearbook 2021 with 83.28% of women registered among the doctors, nurses, pharmacists, health associate professionals and life science professionals working in the healthcare system [42].

Occupation was directly related to exhaustion only in slHCW, while in flHCW, the level of education was a significant determinant. Both items are connected to duties of higher responsibility, which, in the context of uncertainties, procedural changes, shortage of resources and fear of medical errors, generated depletion in the adaptative reserves and, finally, exhaustion. The highest scores of exhaustion were reported by doctors in both groups, although significantly higher in doctors from the flHCW group. Other studies in Romania also showed that doctors were the most affected group by exhaustion [43], in contrast with other countries that generally reported nurses to have the highest scores [35,37,44]. This might reflect a specificity of the Romanian work organization, in which nurses have rather limited autonomy and almost all decisions are made by doctors. This opens the possibility for a preventive intervention in the future based on the re-distribution of the tasks.

Managing the infectious risk in the workplace is essential to decrease stress. Despite the general guidelines that were issued and the infection control measures that were recommended during the stage of the pandemic analyzed in this study, the implementation was specific to each workplace. Management procedures and resources in place were not sufficient to reduce transmission in some medical facilities. In our study, one third of the respondents found the implementation of the safety procedures to be inefficient. The relation between the fear of infection and transmission of the virus to others, exhaustion and burnout was assessed in different studies all around the globe [40,44,45]. It seems that, in some circumstances, pandemic fatigue was present also in medical institutions, with more unprotected exposure to COVID-19 in the third compared to the second wave [29]. The slHCW group was more critical about the risk management efficacy; there was a relation in the univariate analysis with the exhaustion score, but in the final adjusted model, this influence became non-significant.

To the best of our knowledge, this is the largest study on exhaustion among Romanian HCW during the COVID-19 pandemic, despite its limitations. Firstly, the results might not be representative enough of all Romanian HCW; our sample has a better representation of doctors compared to other professions and an imbalanced number of HCW without a university degree; this might have influenced the overall results and does not permit generalization. However, the proportion of doctors and persons with undergraduate studies was similar in the two groups and the comparisons between flHCW and slHCW should not be affected. Secondly, voluntary participation can be a matter of bias in studies focused on mental health issues, but the invitation to participate was addressed at assessing the general impact of the pandemic. From this perspective, the high interest to participate due to personal concerns about psychological issues should not be a main concern. Third, with respect to the confidentiality of the participants, we collected very few personal data. Although the initial emails covered all regions of the country, the location of the respondents was not collected. Therefore, we cannot confirm the geographical representativeness of our results.

## 5. Conclusions

Through this analysis, we showed that there were more similarities than differences between flHCW and slHCW. Even if flHCW had a higher risk of exhaustion, this risk was not negligible in slHCW. Overcommitment and the perception of effort/reward imbalance are the main determinants of exhaustion in both groups of HCW.

Many of the workplace stressors are manageable, but lessons learnt from the previous two COVID-19 waves apparently were not enough to satisfactorily reduce the workload and emotional burden. Nevertheless, the content seemed to be better managed in the flHCW group and the context of the work should attract more attention. For slHCW, the length of working hours was not properly adjusted and a better planning of human resources in this group is needed.

## Figures and Tables

**Table 1:** General characteristics of the population.

Characteristic N (%)	All 1872	flHCW 1311 (70.03)	slHCW 561 (29.97)	*p*-Value
**Demographic characteristics** Gender				0.367
Men	340 (18.16)	245 (18.69)	95 (16.93)	
Women	1532 (81.84)	1066 (81.31)	466 (83.07)	
Age (average ± SD)	48.6 ± 10.9	48.6 ± 11.1	48.8 ± 10.6	0.776 *
Age, years				0.519
20–29	128 (6.84)	89 (6.79)	39 (6.95)	
30–39	240 (12.82)	177 (13.50)	63 (11.23)	
40–49	586 (31.30)	413 (31.50)	173 (30.84)	
>50	918 (49.04)	632 (48.21)	286 (50.98)	
Marital status				0.001
Married/couple	1390 (74.25)	1002 (76.43)	388 (69.16)	
Single	482 (25.75)	309 (23.57)	173 (30.84)	
Education level				<0.001
Undergraduate	179 (9.56)	93 (7.09)	86 (15.33)	
University degree	1693 (90.44)	1218 (92.91)	475 (84.67)	
**Job characteristics**				
Occupation				<0.001
Doctors	1328 (70.94)	1084 (82.68)	244 (43.49)	
Nurses	248 (13.27)	152 (11.59)	96 (17.11)	
Other health professionals	168 (8.99)	64 (4.88)	104 (18.54)	
Health management and support	128 (6.85)	11 (0.84)	117 (20.86)	
Workplace				<0.001
Hospital	368 (19.66)	328 (25.02)	40 (7.14)	
Other	1504 (80.34)	983 (74.98)	520 (92.86)	
Tenure (years)				0.135
<1	37(1.98)	22 (1.68)	15 (2.67)	
1–5	229 (12.23)	160 (12.20)	69 (12.30)	
6–10	163 (8.71)	125 (9.53)	38 (6.77)	
>10	1443 (77.08)	1004 (76.58)	439 (78.25)	
Number of daily working hours (hours)				<0.025
≤8	1556 (83.12)	1073 (81.85)	483 (86.10)	
>8	316 (16.88)	238 (18.15)	78 (13.9)	
Working in shifts				0.0009
No	1524 (81.415)	1047 (79.86)	477 (85.03)	
Yes	348 (18.59)	264 (20.14)	84 (14.97)	
Number of patients/day				
<5		138 (10.53)		
Between 5–15		366 (27.92)		
Over 15		807(61.56)		
Management of risk of COVID-19 infection				0.0002
Very well	647 (34.56)	480 (36.61)	167 (29.78)	
Well	682 (36.43)	492 (37.53)	190 (33.87)	
Acceptable	429 (22.92)	269 (20.52)	160 (22.92)	
Not so well	88 (4.7)	55 (4.2)	33 (5.88)	
Very badly	26 (1.39)	15 (1.14)	11 (1.96)	
**Perceived job stressors**				
Reward score (average ± SD)	5.44 ± 1.54	5.21 ± 1.522	5.99 ± 1.44	<0.001 **
Effort score (average ± SD)	9.19 ± 2.04	9.51 ± 1.89	8.45 ± 2.16	<0.001 **
**Personal factors**				
Overcommitment score (average ± SD)	16.01 ± 2.89	16.34 ± 2.80	15.24 ± 2.94	<0.001 **
COVID-19 diagnosis				0.002
Yes	476 (25.43)	360 (27.46)	116 (20.68)	
No	1396 (74.57)	951 (72.54)	445 (79.32)	
Persistent symptoms of COVID-19				0.023
Yes	201 (46.10)	161 (49.24)	40 (36.70)	
No	235 (53.90)	166 (50.76)	69 (63.30)	
Duration of symptoms				0.138
<1 month	23 (11.44)	19 (11.80)	4 (10.00)	
1–3 months	75 (37.31)	65 (40.37)	10 (25.00)	
>3 months	103 (51.24)	77 (47.83)	26 (65.00)	

* Student’s *t*-test; ** Mann–Whitney U test; Pearson’s chi-squared test otherwise; flHCW: frontline healthcare workers; slHCW: second-line healthcare workers.

**Table 2:** Univariate relation between the determinants and high exhaustion score.

	Total		flHCW		slHCW	
Variables	Coefficient (CI 95%)	*p*-Value	Coefficient (CI 95%)	*p*-Value	Coefficient (CI 95%)	*p*-Value
**Demographics**						
Age	−0.05 (−0.07–−0.03)	<0.0001	−0.06 (−0.08–−0.04)	<0.0001	−0.04 (−0.07–−0.001)	0.04
Gender						
Men	reference					
Women	0.73 (0.18–1.29)	0.010	0.61 (−0.03–1.23)	0.06	12.14 (0.14–2.21)	0.03
Level of education						
Undergraduate	reference					
University degree	1.45 (0.73–2.18)	0.00009	1.09 (0.12–2.08)	0.03	1.14 (0.07–2.22)	0.04
Marital status						
Married/couple	reference					
Single	0.005 (−0.48–0.5)	0.98	−0.52 (−1.11–0.08)	0.09	−0.58 (−1.43–0.26)	0.17
**Objective job characteristics**						
Occupation						
Health management and support	reference					
Doctors	2.77 (1.85–3.68)	<0.001	0.20 (−2.55–2.96)	0.884	1.95 (0.85–3.04)	0.001
Nurses	1.89 (0.84–2.95)	<0.001	−0.53 (−3.38–2.31)	0.712	1.60 (0.29–2.91)	0.017
Other health professionals	1.42 (0.31–2.53)	0.012	−0.94 (−3.91–2.03)	0.534	1.51 (0.27–2.75)	0.017
Workplace						
Other	reference					
Hospital	1.55 (1.10–2.003)	<0.0001	0.64 (−0.15–1.45)	0.11	0.57 (−0.71–1.87)	0.38
Tenure	−0.12 (−0.39–0.15)	0.4	−0.21 (−0.54–0.12)	0.21	0.32 (−0.37–1.02)	0.36
Working h/day						
≤8 h	reference					
>8 h	2.20 (1.76–3.08)	<0.0001	1.64 (0.99–2.29)	<0.0001	3.43 (2.34–4.52)	<0.0001
Night shifts						
No	reference					
Yes	1.02 (0.48–1.57)	0.0003	0.45 (−0.17–1.08)	0.16	2.22 (0.55–1.15)	0.00006
**Perceived job stressors**						
Effort score	1.28 (1.2–1.37)	<0.0001	1.27 (1.16–1.38)	<0.0001	1.24 (1.1–1.39)	<0.0001
Reward score	−1.20 (−1.33–1.075)	<0.0001	−1.17 (−1.33–−1.02)	<0.0001	−1.06 (−1.32–−0.80)	<0.0001
Effort/reward score	3.17 (2.91–3.44)	<0.0001	2.81 (2.51–3.11)	<0.0001	4.39 (3.73–5.03)	<0.0001
Distress (Effort/reward score > 1)	0.25 (0.21–0.28)	<0.0001	4.01 (3.53–4.48)	<0.0001	4.87 (4.07–5.05)	<0.0001
Management of the infection risk in the workplace						
Very well	reference					
Well	0.72 (0.22–1.22)	0.005	0.86 (0.29–1.44)	0.003	0.49 (−0.46–1.44)	0.309
Acceptable	2.10 (1.54–2.67)	<0.001	2.21 (1.53–2.89)	<0.001	2.52 (1.53–3.51)	<0.001
Not so well	2.71 (1.68–3.75)	<0.001	3.11 (1.84–4.38)	<0.001	2.65 (0.95–4.36)	0.002
Very badly	3.34 (1.52–5.16)	<0.001	3.81 (1.46–6.15)	0.001	3.44 (0.65–6.23)	0.016
**Personal factors**						
Overcommitment	1.04 (0.99–1.1)	<0.0001	1.03 (0.96–1.10)	<0.0001	1.01 (0.91–1.12)	<0.0001
HCW diagnosed with COVID-19						
No	reference					
Yes	0.95 (0.47–1.45)	0.0001	0.76	0.009	1.03 (0.07–1.99)	0.003
Persistence of symptoms	reference					
No	1.79 (0.9–2.68)					
Yes		0.00008	1.52 (0.49–2.56)	0.004	2.15 (0.42–3.88)	0.02

flHCW: frontline healthcare workers; slHCW: second-line healthcare workers.

**Table 3:** Regression models of correlation between the risk factors and exhaustion score in flHCW.

Variables	Model 1		Model 2		Model 3		Model 4		Model 5	
	Beta Coef.	*p*	Beta Coef.	*p*	Beta Coef.	*p*	Beta Coef.	*p*	Beta Coef.	*p*
**Demographics**										
Age	−0.144	<0.0001	−0.178	<0.001	−0.169	<0.001	−0.190	<0.001	−0.189	<0.001
Level of education	0.074	0.007	0.048	0.198	0.040	0.234	0.041	0.146	0.059	0.006
**Objective job characteristics**										
Occupation			−0.085	0.003	−0.04	0.12	−0.026	0.23	0.009	0.84
Working h/day			0.160	<0.001	0.098	<0.001	0.043	0.048	0.043	0.046
Number of patients/day										
<5			reference							
5–15			0.007	0.874	−0.0009	0.982	0.003	0.933	0.003	0.919
>15			0.117	0.008	0.046	0.245	−0.005	0.888	−0.005	0.891
**Perceived job stressors**										
Effort/reward score					0.422	<0.001	0.207	<0.001	0.207	<0.001
Management of the risk of infection in the workplace					0.113	<0.001	0.092	<0.001	0.091	<0.001
**Personal factors**										
Overcommitment score							0.532	<0.001	0.531	<0.001
Personal history of COVID-19									0.013	0.519

flHCW: frontline healthcare workers.

**Table 4:** Relation between the variables and high exhaustion score in flHCW.

Variable	Unstandardized Coefficients	Standard Error	Beta	t	*p* *
Age	−0.00566	0.00089	−0.15207	−6.39373	<0.0001
Score of overcommitment	0.05210	0.00388	0.35508	13.42087	<0.0001
Effort/reward score	0.12807	0.01439	0.23497	8.90018	<0.0001
Intercept	−0.54038				

* Regression analysis adjusted for occupation, number or working hours/day, number of patients/day, personal history of COVID-19 and management of the risk of infection; flHCW: frontline healthcare workers.

**Table 5:** Regression models of the correlation between the risk factors and exhaustion score in slHCW.

Variables	Model 1		Model 2		Model 3		Model 4		Model 5	
	Beta Coef.	*p*	Beta Coef.	*p*	Beta Coef.	*p*	Beta Coef.	*p*	Beta Coef.	*p*
**Demographics**										
Age	−0.086	0.04	−0.008	0.048	-0.077	0.038	−0.122	<0.001	−0.1123	<0.001
Gender	0.100	0.02	0.082	0.042	0.075	0.039	0.009	0.777	0.009	0.771
Level of education	0.082	0.05	0.072	0.068	0.042	0.251	0.001	0.733	0.010	0.745
**Objective job characteristics**										
Occupation			−0.124	0.004	−088	0.023	−0.100	0.002	−0.101	0.002
Working h/day			0.246	<0.001	0.158	<0.001	0.064	0.057	0.065	0.053
Night shifts			0.054	0.22	−0.0006	0.880	−0.0004	0.988	0.00006	0.999
**Perceived job stressors**										
Effort/reward score					0.411	<0.001	0.199	<0.001	0.199	<0.001
Management of the risk of infection in the workplace					−0.067	0.08	−0.067	0.04	−0.067	0.04
**Personal factors**										
Overcommitment score							0.521	<0.001	0.523	<0.001
Personal history of COVID-19									0.012	0.701

slHCW: second-line healthcare workers.

**Table 6:** Relation between the variables and high exhaustion score in slHCW.

Variable	Unstandardized Coefficients	Standard Error	Beta	t	*p* *
Average working hours/day	0.04226	0.01500	0.10789	2.81773	0.00501
Score of overcommitment	0.04084	0.00488	0.34874	8.35956	<0.0001
Effort/reward score	0.12349	0.02719	0.18794	4.54216	<0.0001
Intercept	−0.72794				

* Regression analysis adjusted for gender, occupation and nightshift; slHCW: second-line healthcare workers.

**Table 7:** Comparison of the exhaustion scores in frontline healthcare workers and in the control group by occupation.

	flHCW		slHCW		*p* *
	Average + SD	Median	Average + SD	Median	
Doctors	15.023 ± 4.64	15	13.63 ± 4.72	13	0.00002
Nurses	14.28 ± 4.82	14	13.28 ± 5.23	13	0.06
Other health professionals	13.88 ± 3.75	14	13.19 ± 4.37	13	0.20
Health management and support	14.82 ± 6.54	15	11.68 ± 4.16	11	0.11

* Mann–Whitney U test; flHCW: frontline healthcare workers; slHCW: second-line healthcare workers.

**Table 8:** Relation between the high exhaustion scores and the diagnosis of COVID-19 of the HCW in the frontline and the second-line healthcare workers.

	OR (CI 95%)	*p* *
flHCW group		
COVID-19—exhaustion score > 75% of the maximum score	1.36 (1.027–1.82)	0.03
Persistence of symptoms—exhaustion score > 75% of the maximum score	1.41 (0.87–2.31)	0.16
Duration of symptoms—exhaustion score > 75% of the maximum score	1.13 (0.69–1.85)	0.62
slHCW group		
COVID-19—exhaustion score > 75% of the maximum score	1.41 (0.81–2.47)	0.21
Persistence of symptoms—exhaustion score > 75% of the maximum score	2.52 (0.94–6.77)	0.06
Duration of symptoms—exhaustion score > 75% of the maximum score	1.88 (0.54–6.47)	0.31

* Logistic regression; flHCW: frontline healthcare workers; slHCW: second-line healthcare workers.

## Data Availability

Data sharing will take place according to the guidelines established within the Orchestra project. An informed consent agreement for online qualitative research was provided to each participant, who had the option to accept or reject the participation before responding to the first question of the survey. Only subjects who accepted to participate were included in the analysis.
